# *Saccharomyces cerevisiae* Supplementation Enhances Growth and Immune Response in Nile Tilapia During Winter Stress

**DOI:** 10.1155/anu/9955148

**Published:** 2025-09-15

**Authors:** Nantaporn Sutthi, Eakapol Wangkahart, Paiboon Panase, Nanthana Pothakam

**Affiliations:** ^1^Department of Agricultural Technology, Faculty of Technology, Mahasarakham University, Maha Sarakham 44150, Thailand; ^2^Applied Animal and Aquatic Sciences Research Unit, Division of Fisheries, Faculty of Technology, Mahasarakham University, Maha sarakham 44150, Thailand; ^3^Fisheries Division, School of Agriculture and Natural Resources, University of Phayao, Phayao 56000, Thailand; ^4^Unit of Excellence “Physiology and Sustainable Production of Terrestrial and Aquatic Animals” Division of Fisheries, School of Agriculture and Natural Resources, University of Phayao, Phayao 56000, Thailand; ^5^Veterinary, Conservation and Research Section, Animal Management Division, Chiang Mai Night Safari, Chiang Mai 50230, Thailand

**Keywords:** disease resistant, immune response, *Saccharomyces cerevisiae*, winter season

## Abstract

The purpose of this study was to evaluate the effects of baker's yeast (*Saccharomyces cerevisiae*) supplementation on growth, immune-related gene expression, and disease resistance in Nile tilapia (*Oreochromis niloticus*) during the winter season. Fish (an average 5.17 ± 0.33 g) were fed diets containing four different *S. cerevisiae* concentrations: 0 g/kg (control; T1), 5 g/kg (T2), 10 g/kg (T3), and 20 g/kg (T4) for 90 days. The results showed that weight gain (WG) and specific growth rate (SGR) were significantly higher in fish-fed the T4 diet compared to the control group (*p* < 0.05). Additionally, fish-fed the T4 diet showed lower carcass yields but higher fillet yields, along with increased amylase and protease activities (*p* < 0.05). Significant increases (*p* < 0.05) in serum lysozyme activity were found in fish-fed the T4 supplemented diet, and elevated myeloperoxidase (MPO) levels were observed in fish-fed the T3 diet. Moreover, upregulation of *il-8* transcription in the liver was noted in fish feeding *S. cerevisiae* (T2–T4) compared to the control group. In a challenge test against *Streptococcus agalactiae*, survival rates (SRs) were significantly higher in fish-fed the T4 diet compared to the control group (*p* < 0.05). Interestingly, the lowest bacterial counts were recorded in the T3 group (*p* < 0.05). These findings suggest that dietary supplementation with *S. cerevisiae* at 10–20 g/kg enhances growth performance, digestive enzyme activity, immune responses, and disease resistance in Nile tilapia during winter conditions.

## 1. Introduction

Nile tilapia (*Oreochromis niloticus*) ranks among the top 10 most significant species in global aquaculture, with a production exceeding 5.3 million tonnes in 2022 [1]. It is a crucial protein source for low-income populations [2]. In Thailand, a tropical country, tilapia is the most commonly farmed freshwater fish. However, colder winter months lasting 2–3 months in the northern and northeastern regions pose challenges for tilapia farming. Exposure to 13°C has been shown to induce histopathological brain alterations, oxidative stress, and tissue damage in Nile tilapia [3]. Rapid declines in temperature to 13°C trigger physiological stress, impairing functions and elevating cortisol levels, which increase the risk of mortality [4]. Cold-induced metabolic and immune dysfunctions further heighten the susceptibility of tilapia to *Streptococcus agalactiae* infections [4]. Below 10–20°C, reduced feed intake is observed, which slows growth and increases the risk of mortality, particularly under 15°C. Growth has been found to decline by up to 30% between 20 and 22°C, with a lethal threshold occurring at 10–11°C [5, 6].

Temperature is a critical factor affecting growth, metabolism, immunity, and reproduction in Nile tilapia [7]. While most research on thermal stress in fish has focused on rising temperatures due to global climate change [8, 9], increasing frequency of both heat waves and cold snaps [10] may disrupt aquatic ecosystems. Cold spells, though less studied due to their irregular occurrence, can cause mass fish mortality, particularly in tropical and subtropical regions [11]. Cold stress disrupts physiological and hormonal balance, impairing reproductive function by reducing gamete production, altering hormone levels, and decreasing fertility [12]. Prolonged exposure to low temperatures can result in significant mortality, particularly during sudden drops [13]. To sustain tilapia production in regions experiencing seasonal temperature fluctuations, effective cold stress management through optimized energy intake and supportive measures is essential for maintaining health and productivity during winter [14, 15].

Moreover, antibiotics such as oxytetracycline, florfenicol, and trimethoprim/sulfadiazine are frequently used in aquaculture for disease management [16]. The exposure of aquatic organisms to antimicrobials, whether through treatment or chronic sub-therapeutic use, is associated with selective pressure that leads to the emergence of resistant bacterial mutants. This resistance is developed through bacterial DNA mutations and horizontal gene transfer, allowing certain populations to survive antibiotic treatment [17]. As challenges posed by antibiotic-resistant pathogens continue to be encountered in aquaculture, the development of effective alternatives is increasingly emphasized to enhance disease management and sustainability. The rising prevalence of diseases in aquaculture, along with the demand for sustainable production methods, has led to increased interest in probiotics for aquaculture species [18]. Probiotic use is recognized as a promising strategy to improve fish health and reduce reliance on antibiotics, ultimately contributing to more sustainable aquaculture systems.

Baker's yeast, particularly *Saccharomyces cerevisiae*, has been widely recognized in aquaculture as a promising probiotic candidate [19]. It has been demonstrated that supplementing baker's yeast in fish diets has the potential to improve growth, survival, intestinal development, and immune function, especially in fish larvae and juveniles [20]. Yeast is known to be nutrient-dense, containing proteins, vitamins, minerals, and bioactive compounds such as β-glucan, nucleotides, nucleosides, glutamine, peptides, and mannan oligosaccharides (MOS), which are responsible for promoting growth, immune responses, and disease resistance in various aquaculture species [21, 22]. Due to its high protein and nutrient content, yeast is also being investigated as a sustainable alternative to fish meal in aquafeed [23, 24]. Additionally, yeast is involved in cytokine regulation and gastrointestinal health, thereby, supporting fish immune responses [25, 26].

Recent studies have highlighted the beneficial effects of yeast supplementation in mitigating stress in fish. For instance, yeast has been shown to modulate inflammatory responses in Jian carp (*Cyprinus carpio var. Jian*) exposed to biotic stress induced by *Aeromonas hydrophila*, indicating that a suitable replacement level of baker's yeast (1.0%) can be used to prevent excessive inflammation [27]. Moreover, in gilthead seabream (*Sparus aurata*), yeast supplementation has been found to effectively counteract cadmium (Cd) toxicity by reducing Cd accumulation in tissues and restoring normal tissue architecture, thus reversing Cd-induced damage [28]. These findings underscore yeast's role as a protective agent against both biotic and environmental stressors in aquaculture, contributing to enhanced fish health and resilience.

However, the impact of *S. cerevisiae* under winter stress conditions, particularly in relation to growth performance and immune function, remains underexplored. Cold stress is known to present challenges during the winter season, yet limited research has been conducted on this issue. Therefore, further investigation is necessary, and nutritional intervention could be employed to enhance the cold tolerance of fish. This study was designed to address the challenges of rearing Nile tilapia under cold stress during the winter season by supplementing their diet with yeast as a probiotic, serving as an alternative to antibiotics, particularly in response to *S. agalactiae* exposure.

## 2. Materials and Methods

### 2.1. Ethical Statement

The study was conducted in accordance with animal care guidelines approved by the Institutional Animal Care and Use Committee at the Mahasarakham University, Thailand (IACUC-MSU-14/2023).

### 2.2. Experimental Diet Preparation

Commercial baker's yeast (*S. cerevisiae*) was obtained from Perfect (Thailand) at a concentration of 1.5 × 10^9^ CFU/g. The product contained 39.1% crude protein and 1.2% crude lipid by weight. The basal diet used in the study was a commercial feed from Betagro, Thailand, comprising 32% crude protein, 4% crude fat, 6% fiber, and 12% ash (Table 1). The yeast was incorporated into the feed at four different concentrations: 0 g/kg (T1;control), 5 g/kg (T2), 10 g/kg (T3), and 20 g/kg (T4). The supplemented feed was coated with 4% agar, following the use of 20 g of guar gum as a pellet binder, as detailed in our previous study [29]. A proximate analysis of the experimental diets was performed in accordance with the Association of Official Analytical Chemists (AOAC) 2023 standards method [30], with the results presented in Table 2.

### 2.3. Experimental Design

**Figure 1:**
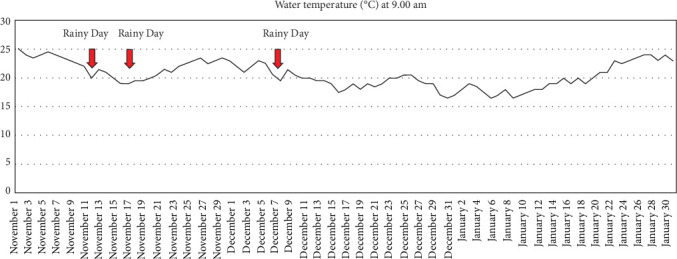
Water temperature (°C) during the 90-day experimental period in the winter season (November to January).

In this study, a completely randomized design (CRD) arrangement of treatments was used. A total of 360 sex-reversed (male) Nile tilapia (*O. niloticus*) were procured from the Maha-Sarakham Inland Fisheries Research and Development Center (Maha-Sarakham, Thailand). The fish were placed in a 50 tonnes concrete tank and subjected to a 2-week acclimation period, during which they were fed a commercial diet containing 32% protein twice daily. Following acclimation, they were transferred to 12 fiberglass tanks, each with a capacity of 1500 liters, with 30 individuals per tank. The fish were then randomly assigned to one of four groups. This experimental design was replicated in triplicate, with the following treatments: T1: fish-fed only a basal diet; T2: fish-fed yeast at 5 g/kg of diet; T3: fish-fed yeast at 10 g/kg of diet; T4: fish-fed yeast at 20 g/kg of diet. The initial mean weight of the fish was recorded as 5.17 ± 0.33 g. Throughout the 90-day experimental period, the fish were fed experimental diets at 5% of their body weight, divided into two daily feedings (09:00 and 16:30). In order to maintain water quality, approximately 80% of the water in the tanks was replaced on a weekly basis. During the experimental period, water quality parameters were measured. The average water temperature (Figure 1) was 20.54 ± 2.24°C (ranging from a minimum of 16.5°C to a maximum of 24.5°C). Dissolved oxygen was 4.81 ± 0.35 mg/L, total ammonia nitrogen (TAN) was 0.23 ± 0.02 mg/L, and pH was 6.70 ± 0.49.

### 2.4. Samples Collections

At the end of the 90-day feeding trial, fish were randomly caught for immune response analysis.

Collection of serum was carried out as our previous described by Van Doan et al. [31]. Briefly, blood samples were immediately collected from the caudal vein after anaesthetization with clove oil (100 mg/L) and transferred into anticoagulant tubes for hematological analysis. Additionally, another blood sample was collected and placed into sterile Eppendorf tubes without anticoagulant for serum collection. The serum was separated from the coagulated blood after incubation at room temperature for 1 h, followed by refrigeration at 4°C overnight. The samples were then centrifuged (5000 rpm) to obtain clear serum, which was securely stored in Eppendorf tubes at −20°C until use in subsequent analyses. Moreover, liver specimens were collected for gene expression. The specimens were dissected and kept in RNAlater (Invitrogen, USA) at 4°C overnight, and then frozen at −20°C until their utilization in subsequent analyses.

### 2.5. Growth Measurements and Economic Analysis

After the trial ended, the growth rate of each fish in all the treatments were recorded at the beginning and the end of the experiment using the mathematical growth model as follows: weight gain (WG; g), average daily gain (ADG; g/day), specific growth rate (SGR; %/day), feed conversion ratio (FCR), and survival rate (SR, %), these model was described by Van Doan et al. [31].

### 2.6. Body Chemical Composition and Organosomatic Indices

After the trial ended, the fillets (*n* = 3 samples/treatment) were used for approximate analysis (crude protein and crude lipid) using AOAC 2023 standards method [30]. In addition, the organosomatic indices (*n* = 6 samples/treatment) such as fillet, carcass, hepatosomatic index (HSI), viscerosomatic index (VSI) and spleenosomatic index (SSI) were calculated as follows by Panase et al. [32].

### 2.7. Blood Chemical and Hematological Analysis

The blood samples were sent to the Veterinary Central Laboratory in Khon Kaen, Thailand, where they underwent a comprehensive biochemical analysis. This included the quantification of aspartate aminotransferase (AST), alanine aminotransferase (ALT), blood urea nitrogen (BUN), and cholesterol levels, as well as the determination of total protein, albumin, and globulin concentrations. These analyses were performed according to the manufacturer's instructions, and the blood examination was conducted following our previous study [31].

Furthermore, hematological evaluations were carried out, including erythrocyte (RBC) and leukocyte (WBC) counts, neutrophil and lymphocyte percentages, hematocrit (Hct) levels, and hemoglobin (Hb) concentrations. Additionally, blood indices such as mean cell volume (MCV), mean cell Hb (MCH), and mean corpuscular Hb concentration (MCHC) were determined according to the established methodology [29].

### 2.8. Antioxidant Enzyme Activity

The superoxide dismutase (SOD) and catalase activity (CAT) in the liver were measured according to Mansour et al. [33] with some modification described by our previous study [31]. The equivalent unit of the activity was determined and expressed in U/g of liver. The serum malondialdehyde (MDA) concentration was determined by measuring thiobarbituric acid reactive substances (TBARS) following the method suggested by our previous study [31].

### 2.9. Immunological Analysis

Serum lysozyme activity was determined by lysing the lysozyme-sensitive Gram-positive bacterium *Micrococcus lysodeikticus*, and the results were expressed as U/mL. A detailed description of the methodology is presented by Van Doan et al. [29]. The myeloperoxidase (MPO) activity in serum was measured using the method described in our previous studies [29], with absorbance recorded at 450 nm. Finally, the serum bactericidal activity and a detailed description of *S. agalactiae* inhibition are presented by our previous study [31].

### 2.10. Intestinal Digestive Enzyme Activities

The intestine samples were collected and homogenized at a ratio of 40 mg of tissue per mL (w/v) in cold Tris–HCl buffer (pH 7.5) using a tissue homogenizer. The homogenate was then centrifuged at 5000 × *g* for 14 min at 4°C to obtain the supernatant, which was used for assessing enzyme activity in the intestinal extract. The activities of digestive enzymes, including amylase, protease, and lipase, were evaluated in the fish intestine as detailed in Wangkahart et al. [34]. Amylase activity was assessed using starch as the substrate, following the procedure outlined by Wang et al. [35]. This method involves the reaction of 3,5-dinitrosalicylic acid with reducing sugars and other reducing compounds, resulting in the formation of 3-amino-5-nitrosalicylic acid. The absorbance of the reaction product was measured at 550 nm using the iMark Microplate Absorbance Reader (Bio-Rad). The amylase activity was measured as 1 mg of maltose produced per min per mg protein. Protease activity was determined using the azocasein assay according to the method of Bezerra et al. [36], with the absorbance measured at 450 nm using the iMark Microplate Absorbance Reader (Bio-Rad). Lipase activity was assessed using the method described by Iijima et al. [37], which employs p-nitrophenyl palmitate (pNPP) as the substrate. The absorbance was measured at 405 nm using the iMark Microplate Absorbance Reader (Bio-Rad). One unit of lipase activity was defined as the release of 1 µmol of pNPP per minute.

### 2.11. Challenge Test

Thirty healthy fish per treatment group were transferred for a challenge study against *S. agalactiae*. Before the challenge test, we determined the LD_50_ using a dose of 0.3 mL of bacterial suspension with concentrations ranging between 1 × 10^8^ and 1 × 10^9^ CFU/mL in normal Nile tilapia. The SRs were 73.33% and 53.33% at these concentrations, respectively. Based on these results, a concentration of 1 × 10^9^ CFU/mL was selected for the challenge test. Each fish in all treatment groups (*n* = 30 samples/treatment) was injected intraperitoneally (i.p.) with 0.3 mL of *S. agalactiae* suspension at a concentration of 1 × 10^9^ CFU/mL. The bacterial suspension was prepared, and the challenge procedure is available in our previous study [38]. Additionally, the relative percentage survival (RPS) was calculated using the formula by Ahmed [39]: RPS = 1− (% test mortality/% control mortality) × 100.

### 2.12. Gene Expression

Four transcripts of cytokines genes, including interleukin-1 beta (*il-1*β), interleukin-8 (*il-8*) and tumor necrosis factor-alpha (*tnf-α*), and one stress-related gene heat shock proteins 70 (*hsp70*) were quantified in liver specimens using beta-actin (*β-actin*) as endogenous control. The primer pairs were shown in Table 3. Liver RNA was extracted according to the manufacturer's protocol the PureLink RNA Mini Kit (Invitrogen, USA). The details of gene expression analysis were determined as described by in previous study [29, 40]. Data calculated for relative expression using the 2^*−*∆∆CT^ method [41].

### 2.13. Economic Analysis

The currency type for economic evaluation in this study is the euro (€) and it was calculated based on 1€ = 36.02 THB (฿). The economic conversion ratio (ECR) and economic profit index (EPI) were calculated using the following equation of Khoklang et al. [42].

  $$\begin{array}{c} \text{ECR }\left(€{\text{ kg of fish}}^{-1}\right)=\text{ FCR }\left(\text{kg of diet}/\text{kg of fish}\right)\times \text{ diet price }\left(€{\text{ kg of diet}}^{-1}\right), \end{array}$$

  $$\begin{array}{c} \text{EPI }\left(€{\text{ fish}}^{-1}\right)=\left[\text{weight gain }\left(\text{kg}\right)\times \text{ selling price }\left(2.221€{\text{ kg}}^{-1}\right)\right]-\left[\text{weight gain }\left(\text{kg}\right)\times \text{ diet price }\left(€{\text{ kg}}^{-1}\right)\right], \end{array}$$

where diet price (€/kg) = ∑ (each feed ingredient × their respective cost [price/kg]/100), and the sale price of fish was estimated at day sale (€/kg).

The price of each diet was determined by multiplying the respective contributions of each feed ingredient by their respective cost per kg. The price of each ingredient (2023 average) was as follows: fish feed commercial = 0.777 €/kg; yeast extract = 0.0410 €/g; guar gum = 0.004 €/g; agar = 0.039 €/g.

Nile tilapia sale price was calculated at selling price = 2.221 (€ kg^−1^), or 80.00 (฿ kg^−1^).

### 2.14. Statistical Analysis

Data were tested for normality and homogeneity of variance before statistical analysis. The data were performed using a one-way analysis of variance (ANOVA) followed by Duncan's post hoc for multiple comparisons among the treatments. The significance was *p* < 0.05. Results were represented as mean ± standard deviation (SD).

## 3. Results

### 3.1. Growth Performances

Table 4 summarizes the effects of *Saccharomyces cerevisiae* yeast supplementation in feed on the growth performance of Nile tilapia over a 90-day winter trial. The T4 group (20 g/kg) exhibited significantly higher in WG, and SGR compared to the control group (*p* < 0.05). However, no significant differences were observed among treatments for FBW, ADG, FCR, or SR (*p* > 0.05). The economic analysis, also presented in Table 4, revealed that ECR ratio tended to increase with higher levels of yeast supplementation. Conversely, EPI ratio decreased with the increasing levels of yeast supplementation in the diet.

### 3.2. Blood Chemical and Hematological Profiles

Table 5 presents the blood biochemical profiles. The albumin levels were significantly higher in the T3 group (10 g/kg *S. cerevisiae* supplementation) compared to the T2 and control groups (*p* < 0.05). No significant differences were observed among treatments (*p* > 0.05) in total protein, globulin, AST, ALT, BUN, or cholesterol. Hematological indices showed that the neutrophil levels of fish in the T3 and T4 groups were significantly higher than those in the T2 group (*p* < 0.05), although no significant difference was observed compared to the control group. Furthermore, no significant differences were noted among treatments (*p* > 0.05) in RBC, WBC, Hb, Hct, lymphocytes, MCV, MCH, or MCHC.

### 3.3. The Body Composition and Organosomatic Indices

The body composition and organosomatic indices are displayed in Table 6. No significant differences were observed in crude protein and crude lipid levels among the treatments (*p* > 0.05). However, fillet values were significantly higher, and carcass values significantly lower, in fish-fed the T4 diet compared to the control group. Additionally, no significant differences were found in the organosomatic indices (HIS, VSI, and SSI) (*p* > 0.05).

### 3.4. Intestinal Digestive Enzyme Activities

Amylase, protease, and lipase activities in fish-fed varying levels of *S. cerevisiae* are shown in Table 7. Dietary yeast supplementation significantly increased digestive enzyme activities compared to the control group (*p* < 0.05). The highest amylase and protease activities were observed in the T4 group, where fish were fed 20 g/kg of yeast, compared to other inclusion levels.

### 3.5. Antioxidant Activity and Immunological Observations

**Figure 2:**
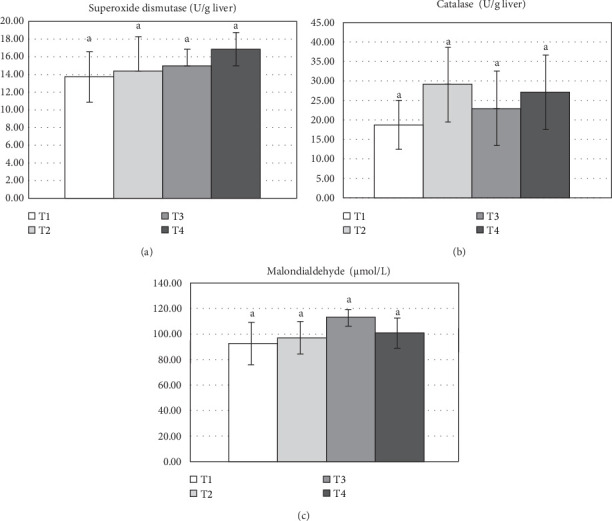
Liver antioxidant activity enzyme and lipid peroxidation, (A) superoxide dismutase (SOD), (B) catalase (CAT), and (C) malondialdehyde (MDA) of Nile tilapia-fed different yeast *Saccharomyces cerevisiae* 90 days under winter stress: T1 (0.0; control), T2 (5 g/kg diet), T3 (10 g/kg diet), and T4 (20 g/kg diet). Data are given as mean ± SD and different lowercase letters indicate significant differences (*p* < 0.05).

**Figure 3:**
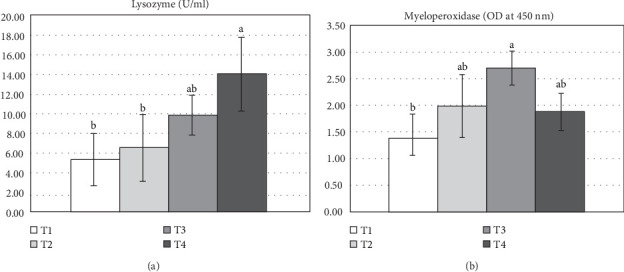
Serum lysozyme activity (LZM) (A) and myeloperoxidase (MPO) (B) of Nile tilapia after fed different yeast *Saccharomyces cerevisiae* 90 days under winter stress: T1 (0.0; control), T2 (5 g/kg diet), T3 (10 g/kg diet), and T4 (20 g/kg diet). Data are given as mean ± SD and different lowercase letters indicate significant differences (*p* < 0.05).

Liver antioxidant activity of Nile tilapia-fed varying levels of *S. cerevisiae* are shown in Figure 2. No significant differences were observed among treatments in liver SOD, CAT, and MDA levels (*p* > 0.05). Serum immunological parameters of Nile tilapia-fed varying levels of *S. cerevisiae* are shown in Figure 3. Results indicated that serum lysozyme activity was significantly higher (*p* < 0.05) in fish-fed the T4 diet compared to the control group (T1) after 90 days of feeding (Figure 3A). Additionally, MPO activity was significantly different (*p* < 0.05) in fish-fed the T3 diet compared to the control group (Figure 3B).

### 3.6. Bactericidal Activity and Challenge Test

**Figure 4:**
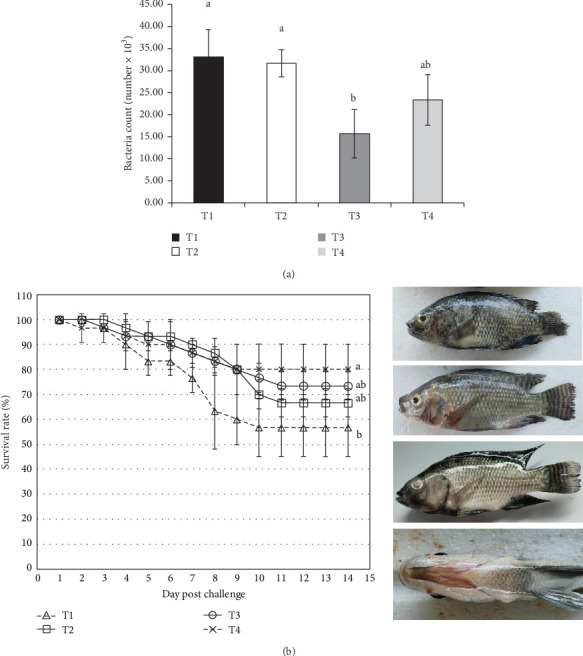
*Streptococcus agalactiae* counts after incubation with serum (A) and percentage of survival rate and clinical signs of Streptococcosis for 14 days post the challenge test with *S. agalactiae* (B) in Nile tilapia after fed different yeast *Saccharomyces cerevisiae* under winter stress: T1 (0.0; control), T2 (5 g/kg diet), T3 (10 g/kg diet), and T4 (20 g/kg diet). Data are given as mean ± SD and different lowercase letters indicate significant differences (*p* < 0.05).

The bacterial count of *S. agalactiae* following incubation with fish serum is presented in Figure 4A. The results indicated a significantly lower bacterial count (*p* < 0.05) in fish-fed the *S. cerevisiae*-supplemented diet at 10 g/kg (T3 diet) compared to the T2 and control groups, while no significant difference was observed compared to the T4 group. Additionally, the SR of postinfection Nile tilapia in the T4 group was significantly higher than that in the control group (*p* < 0.05; Figure 4B); however, no significant difference was observed when compared to the T2 and T3 groups. Mortality in the control group was initially observed on Day 2 postinjection. The clinical signs observed in the affected fish included fin erosion, hemorrhage, dropsy, corneal opacity, and hemorrhagic skin pustules, which appeared on the fourth day postinjection. Furthermore, the mean SRs for control-T1, T2, T3, and T4 groups were 56.67%, 66.67%, 73.33%, and 80.00%, respectively. The highest RPS was observed in fish treated with the T4 diet (53.85%), followed by fish treated with the T3 diet (38.46%) and T2 (23.08%). Fish-fed the diet containing 20 g/kg of the *S. cerevisiae*-supplemented diet demonstrated the highest resistance to *S. agalactiae*.

### 3.7. Gene Expression

**Figure 5:**
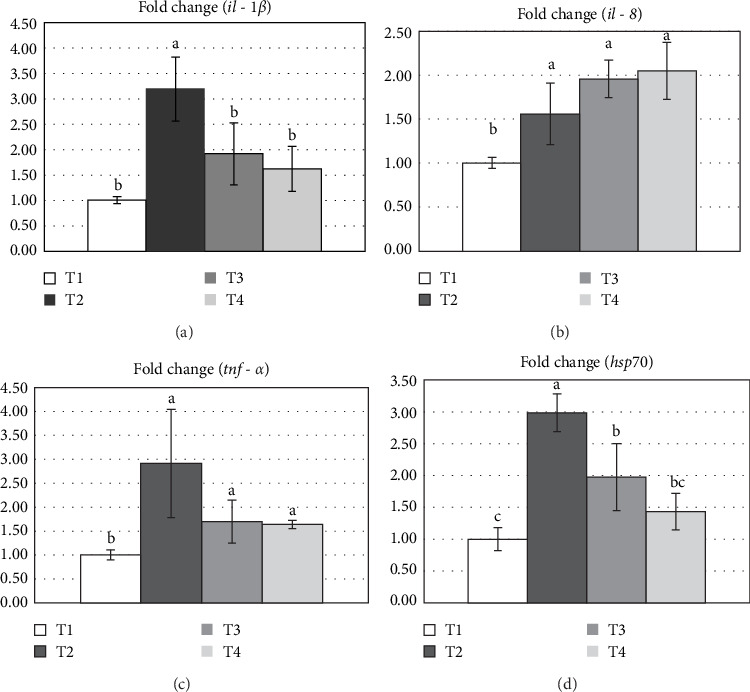
Cytokines genes *il-1*β (A), *il-8* (B), and *tnf-α* (C); and heat shock protein gene (*hsp70*) (D) expression in liver of Nile tilapia after fed different yeast *Saccharomyces cerevisiae* 90 days under winter stress: T1 (0; control), T2 (5 g/kg diet), T3 (10 g/kg diet), T4 (20 g/kg diet). Data are given as mean ± SD and different lowercase letters indicate significant differences (*p* < 0.05).

The effects of *S. cerevisiae* supplementation on the expression of cytokine and hsp genes in the liver of Nile tilapia are shown in Figure 5A–D. Significant upregulation of cytokine genes, specifically *il-8*, was observed in fish-fed with *S. cerevisiae*-supplemented diets (T2, T3, and T4 groups). In contrast, the upregulation of *tnf-α*, *il-1β*, and *hsp70* was observed exclusively in the T2 group (*p* < 0.05).

## 4. Discussion

Temperature is a critical environmental factor that influences the physiological functions, behaviors, and ecological interactions of poikilothermic (cold-blooded) fish species [13]. As ectotherms, fish rely on external temperatures to regulate their body heat, with their thermal tolerance influenced by species, life stage, genetics, and prior thermal experiences [43, 44]. While fish can tolerate a range of temperatures, exposure to extreme heat or cold has been shown to induce sublethal stress or even mortality [43, 44]. The findings of this study demonstrated that juvenile tilapia-fed yeast-supplemented diets during the winter season exhibited significantly improved growth performance, as reflected in higher WG and SGR compared to the control group. Additionally, yeast supplementation was associated with an increase in fillet yield and a reduction in carcass ratio, further highlighting its potential as a dietary intervention. These results contribute to the growing body of evidence supporting nutritional strategies as an effective means to mitigate cold stress in aquaculture. Although research on the role of yeast (*S. cerevisiae*) supplementation in cold-stressed tilapia remains limited, available studies suggest promising benefits. However, most previous investigations have focused on its effects under heat stress. For instance, dietary inclusion of *S. cerevisiae* in tilapia postlarvae resulted in a 16.6% increase in final weight and a 15.1% improvement in SR compared to the control group, along with enhanced resistance to thermal shock [45]. Similarly, tilapia was exposed to acute heat stress (40°C) and supplemented with *S. cerevisiae* exhibited significantly higher SGR than the control group [46]. These findings indicate that yeast supplementation may enhance growth performance under both high- and low-temperature stress conditions, likely due to its high bioavailability and rich nutritional profile [47].

Specifically, yeast contains 45%–49% high-quality protein [48], providing a complete profile of essential amino acids [47] and a rich source of B vitamins [49]. Additionally, yeast extract contains beneficial polysaccharides and carbohydrates, with carbohydrate concentrations ranging between 31% and 51% of the dry biomass [48, 50]. The lipid content of yeast is relatively low (4%–7% of dry biomass) and is predominantly composed of mono-unsaturated fatty acids (MUFAs, 62% of total fatty acids), with palmitoleic acid identified as a major component (2.9%–32%). Polyunsaturated fatty acids (PUFAs) are present in lower concentrations (5.0%–9.7% of total fatty acids), primarily as linoleic acid (4.3%) [48]. Given the high nutritional potential of yeast, its inclusion in the diet may have contributed to the enhanced growth performance observed in this study. In addition to yeast supplementation, several other dietary interventions have been explored to enhance cold tolerance in Nile tilapia. For instance, supplementation with Vitamin C and zinc has been shown to significantly improve immune responses and muscle regeneration under cold stress (15°C), resulting in growth rates comparable to those observed under optimal warm-water conditions [51]. Similarly, dietary supplementation with propylene glycol (PG) in a biofloc system has been reported to enhance winter SRs in Nile tilapia, although its effects on growth performance were not significant [52].

Understanding the role of yeast in enhancing fish digestion is essential, particularly under challenging environmental conditions such as the winter season. The activity of digestive enzymes in fish is highly sensitive to temperature fluctuations and tends to decrease during colder months, resulting in reduced feed intake, impaired nutrient absorption, diminished appetite, and slower growth rates [53, 54]. Prolonged exposure to low temperatures significantly suppresses digestive enzyme activity in juvenile golden pompano and reduces the metabolic rate of the digestive system [55]. Furthermore, basal digestive enzyme secretion is markedly inhibited under such conditions [55]. Environmental and dietary factors also exert a strong influence on digestive enzyme activity in fish. For instance, water acidification (pH reduction from 8 to 7.5) and temperature decreases (from 18 to 2°C) have been shown to reduce intestinal protease activity by up to 56% [56]. Notably, temperature-related responses vary considerably among species, highlighting that digestive efficiency is determined by the interplay between enzyme activity and gut transit time, both of which evolve over extended periods [57]. Dietary supplementation with *S. cerevisiae* has emerged as a promising strategy to mitigate these adverse effects by enhancing digestive enzyme activity. In this study, the inclusion of *S. cerevisiae* (20 g/kg) in fish diets during winter significantly improved the activity of key digestive enzymes, including amylase and protease.

This enhancement facilitated more efficient carbohydrate and protein breakdown, leading to increased nutrient digestibility and improved growth performance. Similar results have been observed that enhanced the activity of lipase, amylase, and protease in tilapia-fed *S. cerevisiae* [58] and *S. cerevisiae*-fermented date palm seed meal [59]. The beneficial effects of yeast are attributed to its probiotic properties and bioactive components, such as yeast cell walls. These components contribute to digestion by secreting extracellular enzymes and supporting the gastrointestinal microbiota, creating a more efficient digestive environment [60]. This interaction fosters synergistic effects, improving nutrient uptake [61] and stimulating the production of bioactive metabolites, including short-chain fatty acids, which nourish the intestinal lining and support gut health [62, 63]. Additionally, yeast cell walls have been shown to stimulate disaccharidase and alkaline phosphatase activity, facilitating the breakdown of carbohydrates into monosaccharides. Improved enzyme activity, particularly maltase and sucrase, enhances nutrient utilization and promotes WG, as demonstrated in broilers [64]. These findings underscore the potential of yeast supplementation to improve nutrient absorption and utilization, even under suboptimal temperatures.

Hematological and biochemical markers are crucial for evaluating fish health, particularly during stress. In this study, the supplementation of *S. cerevisiae* in Nile tilapia during winter was found to enhance immune parameters, including neutrophil count, albumin levels, LZM, and MPO. The observed relationship between neutrophils and MPO can be explained by the role of neutrophils as first-line immune responders that release antimicrobial proteins such as MPO to combat pathogens [65, 66]. These findings reinforce the immune-stimulatory effects of yeast, even under cold stress conditions that typically suppress immune function [67]. The benefits can be attributed to yeast-derived bioactive compounds, such as β-glucans, MOS, and nucleotides. β-glucans, recognized as pathogen-associated molecular patterns (PAMPs), bind to pattern recognition receptors (PRRs), such as Dectin-1 and toll-like receptors (TLRs), activating immune signaling pathways and enhancing cytokine production [68, 69]. Moreover, it has been reviewed by del Valle et al. [19] that the cell wall chitin (poly [1→4]-β-N-acetyl-D-glucosamine) of yeast is identified as a potent immunostimulant that enhances the nonspecific cellular and humoral defenses of various fish species against pathogens. As a probiotic, yeast not only promotes immune responses but also supports overall fish health, making it a valuable addition to aquaculture during challenging conditions. This mechanism was validated in this experiment through bactericidal activity assays and in vivo experiments involving a 14-day challenge with *S. agalactiae*. Fish-fed with yeast demonstrated higher SRs compared to the control group, highlighting the probiotic's role in enhancing tolerance to bacterial infections. These results are reasonable given the mechanisms described above.

Temperature stress is known to impact on the enzymatic antioxidant systems of aquatic animals [70]. Low temperature stress increases endogenous reactive oxygen species (ROSs), including hydroxyl radicals, superoxide, and peroxyl radicals. When ROS production exceeds an organism's capacity to neutralize them, oxidative stress occurs, leading to potential damage to DNA, proteins, and lipids [71, 72]. To counteract this, organisms have developed antioxidant defense mechanisms, such as SOD and CAT [73]. Yeast supplementation may play a role in regulating oxidative stress by modulating signaling pathways [74]. Research has demonstrated that yeast culture can reduce ROS production in PMN cells, thereby protecting cellular systems from oxidative damage [75]. Furthermore, studies suggest that the expression of antioxidant enzyme genes in fish is closely associated with the Nrf2 signaling pathway [76]. The absence of significant differences in SOD, CAT, and MDA levels between Nile tilapia-fed baker's yeast (*S. cerevisiae*) and the control group in present study may be attributed to the yeast's limited intrinsic antioxidant capacity [77]. Alternatively, the antioxidant activity of *S. cerevisiae* could depend on the fermentation biotechnology techniques used during its production [78]. In contrast to red yeast or marine yeasts, which are rich in carotenoids and possess potent antioxidants [31, 77, 79], *S. cerevisiae* primarily functions as an immune modulator. While it plays an indirect role in managing oxidative stress by supporting immune resilience, it does not significantly enhance enzymatic antioxidant defenses such as SOD and CAT. As a result, the antioxidative effects of *S. cerevisiae* were insufficient to modify oxidative damage markers under temperature stress in this study.

Cold shock is known to elevate cortisol levels in fish like tilapia, leading to protein breakdown, immune suppression, and upregulation of stress-related genes such as *hsp70*, *il-1*β, and *tnf-α* [80, 81]. The *hsp70* plays a critical role in stabilizing proteins, supporting immunity, and mitigating inflammation under stress [82]. Its regulation is mediated by heat shock transcription factor 1 (*hsf1*), which governs stress-response pathways and modulates inflammation [83]. Similarly, *il-1*β and *tnf-α*, as proinflammatory cytokines, are crucial for immune regulation, with *il-1*β activating lymphocytes and phagocytes and *tnf-α* being one of the earliest genes expressed during infection [84, 85]. The upregulation of *tnf-α* and *il-1*β during cold stress has been linked to tissue damage and inflammatory responses caused by environmental challenges [86, 87]. In gene expression, our study revealed that Nile tilapia reared during the winter season and fed a diet supplemented with 5 g/kg yeast (T2) exhibited upregulation of *hsp70*, *il-1*β, and *tnf-α* genes. This suggests an immune response to tissue damage caused by cold stress, mediated by yeast's bioactive components. However, higher yeast doses (10–20 g/kg) appeared to dampen these responses, potentially maintaining physiological homeostasis and reducing excessive inflammation. This dose-dependent effect of *S. cerevisiae* aligns with previous research. Yeast has been reported to modulate immune responses by increasing *il-1*β and *tnf-α* while downregulating *hsp70* at specific dosages [69, 88]. At higher doses, yeast supplementation may dampen proinflammatory responses and stress-related gene expression, potentially by maintaining immune balance and mitigating tissue damage. Furthermore, upregulated *il-8* expression was observed in Nile tilapia-fed yeast at all dosages (5–20 g/kg) during the winter season, and higher SRs were demonstrated following pathogen infection. The increase in neutrophil activity with higher yeast supplementation indicates that a direct link exists between dietary yeast and enhanced neutrophil recruitment and activation, which is mediated by *il-8*. As a key chemokine in the inflammatory response, *il-8* is responsible for attracting neutrophils and macrophages to sites of inflammation, thereby, initiating immune defense mechanisms [89]. The β-glucans in yeast likely stimulated *il-8* production, promoting neutrophil activation and migration, which improved pathogen clearance and inflammation resolution [66]. Higher SRs in yeast-fed tilapia may result from this enhanced immune response, as *il-8* binds to CXCR1 and CXCR2, facilitating neutrophil adhesion, migration, and activation [90]. These results suggest that yeast acts as an immunostimulant, amplifying *il-8* expression, and neutrophil-mediated pathogen clearance, thereby, improving disease resistance under cold stress.

The supplementation of baker's yeast (*S. cerevisiae*) in Nile tilapia diets during winter has been demonstrated to provide notable benefits in growth performance, immune function, and disease resistance. However, it was revealed through economic analysis that the increased cost of yeast supplementation resulted in a reduced profit margin. While cold stress mitigation and improved SRs have been effectively achieved through yeast supplementation, its economic viability for large-scale tilapia farming focused on maximizing production remains uncertain. Instead, its application may be more suitable for preserving fish health and reducing natural disease risks under suboptimal environmental conditions. This approach is suggested as a strategic intervention to safeguard stock during challenging periods, although careful consideration of the associated costs is necessary.

## 5. Conclusion

Nile tilapia-fed baker's yeast (*S. cerevisiae*) at 10–20 g/kg showed enhanced immune function, increased digestive enzyme activity, and enhanced resilience, improving tolerance to winter stress. Yeast and its derivatives mitigated the adverse effects of cold temperatures by boosting enzyme activity and nutrient absorption, supporting fish growth and health. However, despite these benefits, the high cost of supplementation led to a significant reduction in profit margins, making it less viable for farms prioritizing profitability. While yeast supplementation can serve as a strategic measure to protect stock during challenging conditions, cost considerations are essential. Future research should focus on cost–benefit analyses, alternative yeast sources, and synergies with probiotics or plant-based supplements to enhance efficacy at lower doses. Additionally, long-term economic studies are needed to balance fish health benefits with farm profitability.

## Figures and Tables

**Table 1:** Analysis of the composition of commercial feed for Nile tilapia and *Saccharomyces cerevisiae*.

Feed analyses^a^	Composition
Crude protein (%)	32
Crude lipid (%)	4
Crude fiber (%)	6
Moisture (%)	12

***Saccharomyces cerevisiae* components^b^**	**Composition**

Crude protein (g/100 g)	39.1
Crude lipid (g/100 g)	1.2

^a^Commercial feed information (Betagro, Thailand). Ingredients of the commercial fish feed: fish meal, soybean meal, corn, broken rice, rice bran, including vitamins and minerals.

^b^Baker's yeast (Perfect, Thailand). Components based on the AOAC (2023) standards method.

**Table 2:** Proximate analysis of the experimental diets (dry matter basis).

Proximate composition (g/100 g)	T1 (control)	T2 (5 g/kg diet)	T3 (10 g/kg diet)	T4 (20 g/kg diet)
Ash	9.73	9.61	9.96	9.66
Crude protein	31.35	31.21	32.38	32.97
Crude lipid	4.38	4.26	4.33	4.68
Crude fiber	4.23	4.42	4.72	4.54
NFE%^a^	50.28	50.48	48.59	48.13
GE (MJ/kg)^b^	17.78	17.73	17.71	17.91

^a^Nitrogen-free extract (NFE%) = 100 − (crude protein + crude lipid + ash + crude fiber).

^b^Gross energy (GE) calculated based on 23.6, 39.5, and 17.2 kJ/g protein, lipid, and carbohydrates, respectively.

**Table 3:** Sequences of primer using in quantitative real-time PCR.

Genes	Primer sequence (5′→3′)	Accession number
*β-actin*	Forward: GCTACTCCTTCACCACCACAG Reverse: CGTCAGGCAGCTCGTAACTC	EF206801
*il-1β*	Forward: TGCACTGTCACTGACAGCCAA Reverse: ATGTTCAGGTGCACTTTGCGG	JF957370
*il-8*	Forward: GCACTGCCGCTGCATTAAG Reverse: GCAGTGGGAGTTGGGAAGAA	NM001279704
*tnf-α*	Forward: CCAGAAGCACTAAAGGCGAAGA Reverse: CCTTGGCTTTGCTGCTGATC	AY428948
*hsp70*	Forward: CATCGCCTACGGTCTGGACAA Reverse: TGCCGTCTTCAATGGTCAGGAT	FJ207463

**Table 4:** Growth performance and economic analysis of Nile tilapia-fed diets supplemented with yeast (*Saccharomyces cerevisiae*) for 90 days during the winter season.

Parameters	T1 (control)	T2 (5 g/kg diet)	T3 (10 g/kg diet)	T4 (20 g/kg diet)
IBW (g)	5.17 ± 0.33	5.36 ± 0.47	5.78 ± 0.66	4.98 ± 0.27
FBW(g)	57.12 ± 2.98	57.81 ± 3.52	62.98 ± 7.18	65.68 ± 3.03
WG (g)	51.62 ± 3.28^b^	52.45 ± 3.35^a,b^	57.20 ± 6.54^a,b^	60.70 ± 3.00^a^
ADG (g/day)	0.57 ± 0.04	0.58 ± 0.04	0.64 ± 0.07	0.67 ± 0.03
SGR (%/day)	2.60 ± 0.12^b^	2.64 ± 0.10^b^	2.65 ± 0.03^b^	2.87 ± 0.07^a^
FCR	1.52 ± 0.14	1.63 ± 0.24	1.60 ± 0.39	1.49 ± 0.16
Survival rate (%)	78.87 ± 5.09	76.67 ± 3.35	81.10 ± 1.90	84.43 ± 5.09
ECR (€ kg^−1^)	1.345 ± 0.123^c^	1.816 ± 0.262^b,c^	2.141 ± 0.523^a,b^	2.661 ± 0.278^a^
EPI (€ fish^−1^)	0.069 ± 0.004^a^	0.058 ± 0.004^b^	0.051 ± 0.006^b^	0.026 ± 0.001^c^

*Note:* Data are given as mean ± SD. Different superscript lowercase letters indicate significant differences (*p* < 0.05). Selling price = 2.221 (€ Kg^−1^).

Abbreviations: ECR, economic conversion ratio; EPI, economic profit index.

**Table 5:** Blood chemical and hematological profiles of Nile tilapia-fed diets supplemented with yeast *Saccharomyces cerevisiae* for 90 days during the winter season.

Parameters	T1 (control)	T2 (5 g/kg diet)	T3 (10 g/kg diet)	T4 (20 g/kg diet)
Blood chemistry				
Total protein (g/dL)	3.00 ± 0.20	3.16 ± 0.15	3.43 ± 0.11	3.40 ± 0.20
Albumin (g/dL)	1.13 ± 0.23^b^	1.20 ± 0.17^b^	1.53 ± 0.05^a^	1.33 ± 0.05^a,b^
Globulin (g/dL)	1.86 ± 0.11	1.96 ± 0.05	1.90 ± 0.17	2.06 ± 0.25
AST (U/L)	95.33 ± 8.38	89.33 ± 5.85	87.66 ± 9.29	90.33 ± 4.50
ALT (U/L)	52.66 ± 17.61	59.66 ± 14.22	47.00 ± 7.81	35.66 ± 3.78
BUN (g/dL)	2.66 ± 0.57	2.67 ± 0.58	3.33 ± 0.57	3.00 ± 1.00
Cholesterol (g/dL)	114.33 ± 6.02	110.66 ± 7.63	116.33 ± 7.63	117.33 ± 3.51
Hematology				
RBC (x10^6^cells/mm^3^)	2.09 ± 0.33	2.10 ± 0.24	2.25 ± 0.35	2.16 ± 0.08
WBC (x10^3^cells/mm^3^)	2.76 ± 1.21	3.16 ± 1.54	3.95 ± 0.51	2.45 ± 0.70
Hemoglobin (g/dL)	7.16 ± 1.38	7.56 ± 1.00	8.13 ± 1.00	7.50 ± 0.95
Hematocrit (%)	36.33 ± 8.08	38.33 ± 2.88	45.33 ± 6.42	42.66 ± 6.42
Neutrophils (%)	11.33 ± 2.08^a,b^	9.66 ± 2.08^b^	15.33 ± 3.21^a^	15.00 ± 1.73^a^
Lymphocytes (%)	84.33 ± 3.78	89.00 ± 1.73	83.66 ± 3.21	84.00 ± 1.73
MCV	172.81 ± 13.96	184.23 ± 14.28	201.89 ± 17.00	197.09 ± 23.48
MCH	34.17 ± 1.51	36.16 ± 0.63	36.22 ± 1.61	34.66 ± 3.42
MCHC	19.81 ± 0.75	19.72 ± 1.89	17.98 ± 0.85	17.61 ± 0.48

*Note:* Data are given as mean ± SD. Different superscript lowercase letters indicate significant differences (*p* < 0.05). RBC, erythrocyte; WBC, leukocyte.

Abbreviations: ALT, alanine aminotransferase; AST, aspartate aminotransferase; BUN, blood urea nitrogen; MCH, mean cell hemoglobin; MCHC, mean corpuscular hemoglobin concentration; MCV, mean cell volume.

**Table 6:** The body composition and organosomatic indices of Nile tilapia-fed diets supplemented with yeast *Saccharomyces cerevisiae* for 90 days during the winter season.

Parameters	T1 (control)	T2 (5 g/kg diet)	T3 (10 g/kg diet)	T4 (20 g/kg diet)
Crude protein (%)	72.55 ± 2.16	74.22 ± 1.30	73.85 ± 1.31	74.44 ± 1.14
Crude lipid (%)	2.86 ± 0.54	2.87 ± 0.37	3.10 ± 0.53	2.87 ± 0.54
Fillet (%)	34.38 ± 2.33^b^	35.57 ± 3.29^b^	38.06 ± 2.26^b^	43.23 ± 0.76^a^
Carcass (%)	53.70 ± 2.17^a^	51.64 ± 3.06^ab^	49.42 ± 0.49^bc^	46.86 ± 1.52^c^
HIS (%)	1.93 ± 0.22	1.39 ± 0.53	2.24 ± 0.51	1.70 ± 0.05
VSI (%)	10.11 ± 0.19	10.94 ± 1.23	10.22 ± 1.61	8.33 ± 0.75
SSI (%)	0.28 ± 0.04	0.23 ± 0.03	0.29 ± 0.05	0.27 ± 0.01

*Note:* Data are given as mean ± SD. Different superscript lowercase letters indicate significant differences (*p* < 0.05).

Abbreviations: HIS, hepatosomatic index; SSI, spleenosomatic index; VSI, viscerosomatic index.

**Table 7:** The intestinal digestive enzyme activities in Nile tilapia-fed diets containing the yeast *Saccharomyces cerevisiae* for 90 days during the winter season.

Parameters	T1 (control)	T2 (5 g/kg diet)	T3 (10 g/kg diet)	T4 (20 g/kg diet)
Amylase (U/mg)	2.52 ± 0.04^b^	2.55 ± 0.03^ab^	2.56 ± 0.01^a^	2.57 ± 0.01^a^
Protease (U/mg)	19.97 ± 0.05^b^	20.01 ± 0.08^b^	20.03 ± 0.03^b^	20.10 ± 0.05^a^
Lipase (U/mg)	54.13 ± 2.79	54.07 ± 1.11	54.52 ± 3.77	55.07 ± 1.07

*Note:* Data are given as mean ± SD. Different superscript lowercase letters indicate significant differences (*p* < 0.05).

## Data Availability

The data that support the findings of this study are available from the corresponding author upon reasonable request.
